# Green synthesis of metalloid nanoparticles and its biological applications: A review

**DOI:** 10.3389/fchem.2022.994724

**Published:** 2022-09-26

**Authors:** Arpita Roy, Shreeja Datta, Ritika Luthra, Muhammad Arshad Khan, Amel Gacem, Mohd Abul Hasan, Krishna Kumar Yadav, Yongtae Ahn, Byong-Hun Jeon

**Affiliations:** ^1^ Department of Biotechnology, School of Engineering and Technology, Sharda University, Greater Noida, India; ^2^ Delhi Technological University, Delhi, India; ^3^ Department of Chemical Engineering, College of Engineering, King Khalid University, Abha, Saudi Arabia; ^4^ Department of Physics, Faculty of Sciences, University 20 Août 1955, Skikda, Algeria; ^5^ Civil Engineering Department, College of Engineering, King Khalid University, Abha, Saudi Arabia; ^6^ Faculty of Science and Technology, Madhyanchal Professional University, Bhopal, India; ^7^ Department of Earth Resources and Environmental Engineering, Hanyang University, Seoul, South Korea

**Keywords:** nanoparticles, metalloids, green materials, bioactive compounds, applications

## Abstract

Synthesis of metalloid nanoparticles using biological-based fabrication has become an efficient alternative surpassing the existing physical and chemical approaches because there is a need for developing safer, more reliable, cleaner, and more eco-friendly methods for their preparation. Over the last few years, the biosynthesis of metalloid nanoparticles using biological materials has received increased attention due to its pharmaceutical, biomedical, and environmental applications. Biosynthesis using bacterial, fungal, and plant agents has appeared as a faster developing domain in bio-based nanotechnology globally along with other biological entities, thus posing as an option for conventional physical as well as chemical methods. These agents can efficiently produce environment-friendly nanoparticles with the desired composition, morphology (shape as well as size), and stability, along with homogeneity. Besides this, metalloid nanoparticles possess various applications like antibacterial by damaging bacterial cell membranes, anticancer due to damaging tumour sites, targeted drug delivery, drug testing, and diagnostic roles. This review summarizes the various studies associated with the biosynthesis of metalloid particles, namely, tellurium, arsenic, silicon, boron, and antimony, along with their therapeutic, pharmaceutical and environmental applications.

## Introduction

Nanotechnology has revolutionized in different areas of science, ranging from electronics to environmental as well as medicine. It involves the production of nanomaterials at the nanoscale level, wherein nanoparticles are a class of materials with less than 100 nm dimension. The role of nanomaterials in the area of nanomedicine has shown various promising promotions in a broad range of health-related issues like diagnostic as well as therapeutic, environmental problems like pollutants and degrading soil quality, and so on. Therefore, nanomaterials are a useful approach for curing infections and improving survival rates amongst patients, and as a result open a newer promising window.

Chemical elements which fall between metal and nonmetals categories are known to be metalloids. As a result, they are difficult to be classified as either one of them. There is not a standard definition or agreement for a metalloid with respect to which they could be classified in a correct manner. Metalloids featuring metals as well as nonmetals are known to be semimetals (Marzo and La Mendola, 2021). Metalloids generally have several effects on the cells as well as tissues, further being efficient in regard to therapeutics—for instance, the role of arsenic in the treatment of syphilis prior to antibiotic discovery in current Food and Drug Administration’s approval, that is FDA’s approval for arsenic dioxide as a drug for the treatment of acute promyelocytic leukemia (Hughes et al., 2011). Arsenic exists in two oxidation states which are biologically crucial, As (III) as well as As (V), but its function as a carcinogen and therapeutic is still an argumentative concern (Mudhoo et al., 2011). Even though the efficiency of some of these elements as a potential drug candidate is vague, proof for the same as curatives is promising. Metalloid nanoparticles are generally utilized for diagnostic assessments; for example-cadmium-telluride nanoparticles along with polymer coating of bismuth sulfide are used in the form of quantum dots for *in vivo* imaging as well as computed tomography (CT). In comparison with the conventional imaging agents (iodinated) for CT assays, exceptional stability in higher concentrations, longer circulation times (>2 h) *in vivo*, high efficacy and safety profile, and higher X-ray absorption (at least fivefold finer than iodine) make this bismuth sulfide nanoparticles a better alternative (Rabin et al., 2006). Besides this, antimony has also been efficiently implemented as a treatment alternative of parasitic diseases like leishmaniasis as well as schistosomiasis (Herrera-Martínez et al., 2020). Tellurium, on the other hand, affects thiol redox associated activity. Chemical bond between Te (IV) and thiols or formation of a disulfide bond in any of the specific proteins leads to conformational changes, and thus might further result in the loss of protein-like inactivation of cysteine proteases (Cunha et al., 2009). Silicon dioxide has also been seen as a targeted drug delivery carrier (Xu et al., 2006). Boron derived nanoparticles are used in cancer as well as neutron capture therapy (Byrappa et al., 2008). There exist two preparation methods for synthesizing metalloid nanoparticles-top- down and bottom-up synthesis. The former employs a destructive approach, starting from a larger molecule and further decomposing it into smaller parts, further getting converted into appropriate nanoparticles. This method of synthesis involves grinding, milling, physical vapor deposition, as well as other decomposition techniques (Iravani, 2011). The latter involves a reverse approach where NPs are produced from relatively simpler substances, also known as the building up approach. This approach includes sedimentation as well as reduction techniques like sol-gel, spinning, biochemical synthesis, and so on (Iravani, 2011). Although, both mentioned synthesis techniques have problems associated with them, i.e., costly, low throughput, slow process, and not easily scalable, and uncontrolled synthesis. As a result, the use of biological materials for nanoparticle synthesis has attracted a lot of researchers due to their feasibility as well as less toxicity (Khan et al., 2017). In this review an overview of various studies associated with the biosynthesis of metalloid particles using bacteria, fungi and plant has been discussed. Further various application of metalloid nanoparticles has been discussed.

## Mechanism of metalloid nanoparticles synthesis

The process of metalloid nanoparticle synthesis involves various steps including selection of precursor material, synthesis method, centrifugation, separation, and production of NPs. Initially, the precursor material is taken from biological sources like microbial based: either bacteria (such as *Bacillus selenitireeducen*, *Sulfurospirillum barnesii*, etc) or fungi (like *Saccharomyces cerevisiae, P. chrysogenum*, etc), or plant-based extracts (like extracts of *P. ginseng*, bamboo, etc). The biological extract is prepared for further undergoing different biosynthesis techniques using different methods like exfoliation, microwave assisted approach, synthesizing intracellularly, pulverization, among others, wherein different chemicals or agents are used which react with the biological extract. After this, the solution changes its colour indicating the success of the reaction between the agents and therefore further production of NPs. Extracts are then centrifuged where the supernatant is discarded or collected (for different purposes) and the pellet is separated out to obtain nanoparticles. This pellet is finally washed with ethanol or water and dried for procuring the final metalloid nanoparticles. These nanoparticles are then subjected to characterization.

## Characterization methods used for determining metalloid nanoparticles synthesis

**FIGURE 1:**
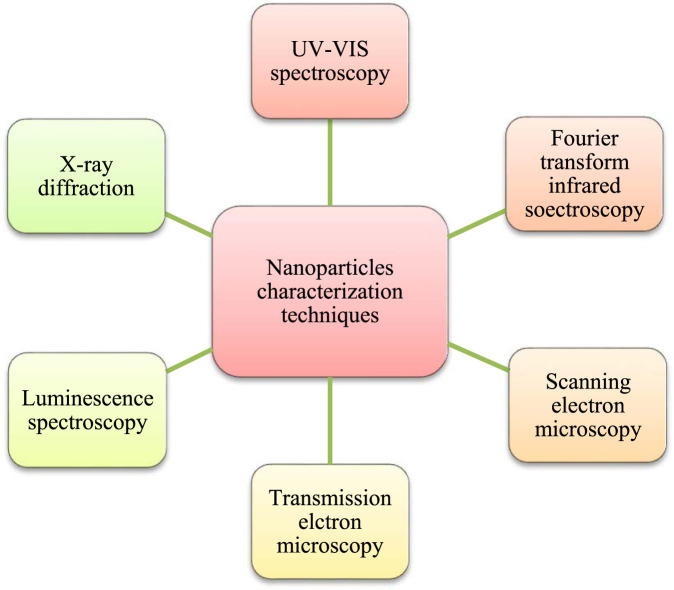
Different characterization techniques for metalloid nanoparticles.

Metalloid nanoparticle characterization is required for correlating their physicochemical characteristics with their toxicity as well as biological impacts (Piacenza et al., 2018). Initially, their physicochemical characterization was conducted *via* a lot of routine lab techniques for analyzing their morphology (like shape and size), porosity, dispersion pattern, surface chemistry, and crystallinity (Kapur et al., 2017). The most common characterization techniques are UV-VIS spectroscopy, X-ray diffraction (or XRD), luminescence spectroscopy, Fourier transform infra-red spectroscopy (or FTIR), scanning electron microscopy–energy dispersive X-ray spectroscopy (or SEM-EDX) and transmission electron microscopy (or TEM) (Figure 1). XRD detects NP’s lattice structure, crystallite size as well as crystallinity with the use of Debye–Scherrer equation (ArmijoGarcia et al., 2020). TEM and SEM determine the morphology of NPs by deducing their size distribution and elemental composition (Estevam et al., 2017; ArmijoGarcia et al., 2020). Kapur (Kapur et al., 2017) studied the magnified field emission scanning electron microscopy whose images provided information about the composition as well as the nature of NPs. Besides this, FTIR provides reproducible analyses to reveal the functional groups present on the surface of NPs. These groups might also be involved in metal ion reduction and NPs capping, thus ensuring the colloidal stability (Wadhwani et al., 2017; Rahman et al., 2019). Dynamic light scattering (DLS) provides NPs hydrodynamic diameter as well as a good insight into their aggregation *via* measuring their Brownian motion, apart from determination of the surface charge (z-potential) of the NPs (Kapur et al., 2017). Atomic force microscopy (AFM) determines the quantitative information regarding the length, surface texture, width, and height of NPs *via* tridimensional visualization (MohammedSafwat and ET., 2013).

## Biosynthesis of metalloid nanoparticles

**FIGURE 2:**
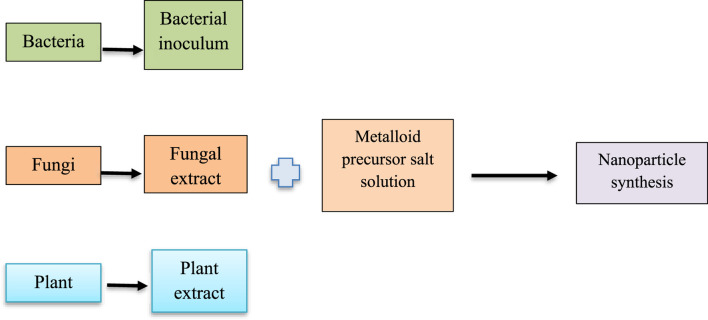
Biosynthesis of Metalloid nanoparticles.

Biosynthesis of metalloid nanoparticles (Figure 2) includes the role of biological agents leading to progress in various fields like biological sciences, engineering, and chemistry (Li et al., 2011; Pugin et al., 2014). Their production by chemical methods is usually inefficient due to high costs as well as toxic and harmful waste generation. As a result, biogenic ways for nanoparticle production are termed as a “green” or environmentally friendly procedure due to their less energy requirement in comparison to chemical methods (Bhattacharya and Mukherjee, 2008).

Studies have proved that metalloid nanoparticles are mostly produced by bacteria as well as microscopic fungi; mainly pathogenic. A comparative study on metalloid nanoparticles synthesised from different biological agents has been mentioned in Table 1.

**TABLE 1:** A comparative table for metalloid synthesised from different biological agents.

Agents	Scientific names	Nanoparticles	Part used	References
*Bacteria*	*Bacillus selenitireducens*	Tellurium	-	Iravani, (2014)
	*Sulfurospirillum barnesii*	Tellurium	-	Baesman et al. (2007)
	*Bacillus* sp	Tellurium		Zare et al. (2012)
	*Actinobacter* sp	Silicon		Singh et al. (2008)
	*S. marcescens*	Antimony		Bahrami et al. (2011)
*Fungi*	*Saccharomyces cerevisiae*	Antimony	-	Jha and Prasad, (2009)
	*Fusarium oxysporum*	Silicon	mycelia	Bansal et al. (2005)
	*P. chrysogenum*	Tellurium		Barabadi et al. (2018)
	*A. pullulans*	Tellurium		Liang et al. (2019)
	*M. humilis*			
*Plant*	*D. alata*	Antimony	tubers	Indurkar et al. (2019)
	bamboo culms	Silicon	Stem	Vinay et al. (2016)
	*Panax ginseng*	Boron	roots	Deshmukh et al. (2019)
	*Morus nigra*		Leaves	
	*Hovenia dulcis*		stems	
	Lime, orange, and lemon	Tellurium	Fruits	Medina et al. (2019)

### Bacterial synthesis

Bacteria possess the exceptional ability of reducing metalloid ions for nanoparticle synthesis. Some of the bacterial species have developed the capability of retreating certain defense mechanisms for checking stresses such as toxicity of metalloid ions or metals. The potential of bacteria for its survival and growth in stressful situations is because of specific resistance mechanisms like efflux pumps, inactivation as well as complexation of metals or metalloids, impermeability and absence of specific transport systems, alteration of solubility and toxicity *via* redox state changes of ions, extracellular precipitation and lastly, volatilization of toxic metals or metalloids due to enzymatic reactions (Reygaert, 2018). Besides this, there exist various drawbacks associated with bacterial synthesis including tedious purification steps as well as ineffective understanding of the procedures involved. The significant challenges with respect to this are operating shape and size of NPs and obtaining monodispersity. Another challenge involves the scaling up of processing at the production level. Moreover, not much is known regarding the mechanistic features as well as information in this respect, which is required for the economic as well as rational growth of NP biosynthesis.

Iravani (2014) conducted an experiment for studying the production of tellurium nanoparticles using anaerobic bacteria, *Bacillus selenitireducens* as well as *Sulfurospirillum barnesii*. When *B. selenitireducens* is used, nanorods with 10 nm diameter are formed initially, further leading to the formation of 200 nm length nanorods which are congregated together for forming a bigger rosette of around 1000 nm. On the other hand, *S. barnesii* was responsible for the generation of small and extracellular nanospheres, irregular in shape and with a diameter less than 50 nm (Baesman et al., 2007). One more study revealed that *Bacillus* sp., transformed tellurium, were extracted from northern Iran (the Caspian Sea) and was involved in the intracellular biosynthesis of these NPs. They were generated *via* liquid nitrogen as well as purified using a water extraction system containing n-octyl alcohol. Transmission electron microscopy displayed nanoparticles having rod shape of about 20 × 180 nm dimensions, with a hexagonal crystalline structure (Zare et al., 2012).

Singh et al. (2008) examined the role of *Actinobacter* sp. for silicon NP production. Bacterial species were extracted in the form of an impurity from the flask having potassium ferrocyanide as well as potassium ferricyanide (Bharde et al., 2005; Bharde et al., 2008). This bacterial colony was inoculated and afterwards incubated for a day under 200 rpm at 27°C. Furthermore, these bacterial cells were collected as well as rigorously washed using autoclaved water in the presence of sterile conditions. This bacterial biomass (1 g wet weight) was then suspended again in the aqueous solution (100 ml) of 1 × 10^−3^ M K_2_SiF_6_. The reaction of bacterial biomass along with solutions of SiF6^2−^ was carried out for 48 h and the products were harvested in a sterile state *via* centrifugation. The mixture of products obtained from the K_2_SiF_6_ reaction was examined using TEM. Particles which were implanted in the matrix were quasi-spherical morphologically with 10 nm. Apart from this, electron diffraction analysis of these silica nanoparticles showed the crystalline nature of these particles. Silicon nanocomposites also displayed well-defined Bragg reflections suggesting evidence for their crystalline nature.

Antimony NPs were also synthesized using bacterial isolates extracted from the Caspian Sea involving biomolecule manufacturing processes known as solid state fermentation (Bahrami et al., 2011). In the initial step, antimony associated NA plates (SbCl_3_, 1% w/v) with bacterial isolates were inoculated. After inoculation, NA media underwent incubation in aerobic conditions at 30°C. Post 72 h, the collected cells were suspended in normal saline and further washed with distilled water by centrifugation for 10 min. Cell pellets were then transferred into a mortar where liquid nitrogen was added, leading to freezing of these pellets and further disruption by a pestle. The resultant slurry with antimony sulfide NPs as well as cell debris were suspended in a two-phase system having 1:2 ratio of n-octyl alcohol to distilled water and shaken vigorously. Two of these phases were mixed and fully segregated through centrifugation of 15 min, following which these biosynthesized NPs were clearly observed in the bottom. These settled NPs were then suspended again in a mixed system comprising water, chloroform, and ethyl alcohol (4:3:1). They were then harvested and put through further evaluation. This study included *S. marcescens* for the formation of antimony NPs. Formed NPs were generated as well as extracted *via* two-solvent phase partitioning system and TEM analysis indicated the sizes of NPs were less than 35 nm.

### Fungal synthesis

Fungi contains a wide range of secondary metabolites which act as a reducing agent in nanoparticles synthesis (Roy et al., 2021). Biosynthesis of nanoparticles usually occurs by edible as well as medicinal basidiomycetes, since they are broadly utilized in food additives and pharmaceuticals; they are neither toxic nor pathogenic, they own efficient enzyme systems and able to generate larger quantities of biomass (Roy et al., 2022). Xylotrophic basidiomycetes like *Lentinus edodes*, *Grifola frondosa*, *Ganoderma lucidum,* and *Pleurotus ostreatus* are used for culture preparation and can be distinguished through easy as well as safe culture, assessed as high-quality fungi (edible) and as enzyme promoters. The potential of these basidiomycetes in regard to producing nanostructures involves the procedure of synthesis, morphological attributes, and nanoparticle localization, which are yet not well researched and understood in comparison to that of lower fungi as well as bacteria. Additionally, most of the research done in this area used *in vivo* fungal cultures, unpurified fungal media filtrates with various unidentified enzymes as well as additional substances, or partly refined enzymes (Sanghi et al., 2011; Durán et al., 2014; Lateef and Adeeyo, 2015). Simultaneously, biosynthesis of NPs with the assistance of pure enzyme preparations isolated from fungal extracts has not been practically considered yet. Different studies for several enzymes derived from basidiomycetes have not been carried out, even though it is feasible that various enzymes retrieve NPs by distinct means. With respect to this, it is significant to regulate more extensive examinations for biological entities along with their metabolites for efficient NP synthesis of preferable size, composition, and shape along with the required strength and uniformity too. Biological NP synthesis *via* medicinal xylotrophic fungi is therefore a crucial subject accounting for the environmental safety, clarity, and availability of this method. In addition to this, another reason for the consideration of fungi for NPs production is their ability of secreting extracellular enzymes and proteins, as well as their economic liability. In comparison to bacteria, fungal biomass has an advantage when used for NPs biosynthesis because of its filamentous structure (Guilger-Casagrande and Lima, 2019). Correspondingly, it is essential to fully study as well as examine the ability of fungal agents and their metabolites for metalloid NP biosynthesis with different chemical compositions.

Jha and Prasad (2009) studied the production of antimony NPs *via* a biosynthesis method involving yeast as an initial material. *Saccharomyces cerevisiae* was selected for studying its potential as a promising fungal candidate for the synthesis of antimony trioxide (Sb_2_O_3_) NPs. The initial culture was produced by letting the fungus grow in the form of a suspended culture having carbon as well as nitrogen sources for one and a half days. After this, 25 ml of the solution was filtered and further diluted *via* the addition of 30% ethanol. This culture after dilution was then grown for another day till the time a light strawish color appeared. After growing for one more day, 20 ml of antimony trichloride (SbCl_3_) (0.025 M) was added and then the mixture was heated for 10–20 min at 60°C till the time white precipitate was observed. This suggested the formation of antimony NPs synthesis. TEM analysis further showed that these nanosized particles were almost spherical-shaped and size ranging between 2–10 nm.

Another study was conducted on the culture of *Fusarium oxysporum*, a pathogenic fungus affecting plants (Bansal et al., 2005). This fungal mycelium was inoculated along with the MGYP medium (around 100 ml) and then incubated for 3 days at 200 rpm in the presence of shaking at 27°C. Fungal mycelia was collected thereafter and washed thoroughly after incubation in a sterile state. After this, 20 g of wet weight of fungal substrate was suspended again in aqueous solution of 1023 M K_2_SiF_6_ (at pH 3.1). The reaction of fungal biomass with the SiF6^2−^ ions resulted in the generation of products which were harvested in the presence of sterile conditions at different time intervals by separating fungal mycelia from the aqueous extract *via* filtration. These products were silica-based NPs. Furthermore, 20 g of wet weight of fungal substrate when reacted with aqueous solution (around 100 ml) of 10^−3^ M, K_2_SiF_6_ gave rise to 34.2 mg of silica-based NPs after drying them to powder. Apart from this, FTIR analysis indicated a resonance at *ca.* 1086 cm^−1^. This strong band proves the excited Si–O–Si stretching mode of vibration which was antisymmetric (Innocenzi et al., 2003) and absence of the same from the spectrum of pure K_2_SiF_6_. For the biosynthesis of tellurium-based NPs by fungal agents, various studies were conducted. Barabadi (Barabadi et al., 2018) studied *P. chrysogenum* PTCC 5031 which was harvested along with 21 g sucrose as well as 3 g yeast extract in distilled water (around 1000 ml) at 120 rpm for 7 days at 28°C. This culture was then subjected at 10,000 rpm for 10 min for separation of mycelia from the supernatant. After this, **1.0××10** ^**−3**^ **mol/L** of K_2_TeO_3_, 3H_2_O was prepared and sustained at sterile conditions post autoclaving, after which 100 ml of potassium tellurite solution was also added to the supernatant (100 ml) at a pH value of 9 and lastly, incubated at 120 rpm for 5 days (overall concentration of K_2_TeO_3_, 3H_2_O being 0.5**××10** ^**−3**^ **mol/L**). Thereafter, biofabricated tellurium based NPs were produced and centrifuged at 15,000 rpm for 15 min for their separation from the reaction medium. Eventually for enhancement of purification of these, the settled NPs were washed three times by deionized double distilled water and thereafter dried at around 40°C along with storage in vials for further characterization. Deduction of NP development was noticed not just by the change of colour (pale yellow to dark), but also from Tyndall effect (King, 2016), indicating their nanoforms. In addition to this, the SEM graph also revealed that they were sphere-like in shape and properly distributed in the solution, having 33.80 nm as their diameter. Liang (Liang et al., 2019) proved the synthesis of tellurium based NPs from various fungal agents. Most of them were able to develop on malt extract agar (MEA) altered with 1.0×10^−3^ mol/L sodium tellurite (Na_2_TeO_3_), expressing various shades of black colouration. Toxicity of the solution was more prominent at 5.0×10^−3^ mol/L concentration, as suggested by decreased colony expansion rates as well as black colouration degree. The impact of Na_2_TeO_3_ on *A. pullulans* as well as *P. glomerata* was powerful at both 1.0×10^−3^ mol/L and 5.0×10^−3^ mol/L concentrations. NPs produced from *A. pullulans*, *P. glomerata*, *M. humilis* as well as *T. harzianum* post growth with Na_2_TeO_3_ had a broad range of shapes and sizes. Particles generated on surfaces of *A. pullulans* as well as *M. humilis* were granular; those generated from *M. humilis* were small but formed aggregates; those from *A. pullulans* were also similar in size. NPs produced from *T. harzianum* as well as *P. glomerata* were pillar as well as needle shaped with varying sizes. Low concentrations of NPs were detected in fungal supernatants grown along with 1.0×10^−3^ mol/L Na_2_TeO_3_ with most of their diameters ranging from 40 to 70 nm and between 80 and 200 μg L−1 concentrations. *P. glomerata* generated maximum NPs at concentrations >200 μg L^**−1**^, while *A. pullulans* generated least at 79 μg L^**−1**^. The concentrations of NPs of *M. humilis* as well as *T. harzianum* were 95 μg L^**−1**^ as well as 174 μg L^**−1**^respectively.

### Plant based synthesis

Extracts of the plant comprised flavonoids, flavones, alkaloids, apigenin, luteolin as well as ascorbic acid (Ali et al., 2017; Roy and Bharadvaja, 2017; Roy, 2017; Roy and Bharadvaja, 2019; Raina et al., 2020; Roy, 2021). Generally, these phytocompounds can be converted into their derivative quinone forms when reactive sites are present. They even have abundant potential for exfoliation and stabilization of layered materials (Roy, 2021). They have been a new topic for plant research as they act as reducing as well as stabilizing or capping agents (Kumar and Yadav, 2009; Borase et al., 2014). Apart from these, other phytochemicals like polyphenols, quinols, flavones, chlorophyll pigments, terpenoids, and alkaloids are also being considered for metal ion bioreduction as well as stabilization of NPs (Huang et al., 2011). Plant based synthesis of NPs is a cost-effective as well as eco-friendly option where the extract of the plant is utilized both as a stabilizing as well as reducing agent for metalloid NP synthesis. This method of synthesis involves non-toxic reactions, as a result, maintaining mild conditions for the reaction (Rai and Posten, 2013). Based on the current engineered materials, it is not wrong to mention that plants have the potential of producing a broad range of nanostructures. Reduction in these reactions is caused by plant metabolites like alkaloids, catechins, phenolics, terpenoids, and flavonoids) (Mohammadinejad et al., 2016).

Indurkar et al. (2019) selected tubers of *D. alata* for synthesizing antimony-based NPs, wherein the tubers were washed, peeled, and dried, further undergoing pulverization. This pulverized powder was then stored at 27°C. Plant extract was prepared by adding this powder into 100 ml deionised water, then kept in magnetic stirring for 10 min, and further transferred into a preheated water bath at 80°C as well as being incubated for 15 more minutes. The next step was to centrifuge the supernatant for 15 min at 8,000 rpm. For the actual synthesis of the NPs, 0.456 g antimony trichloride (0.1 M) was dissolved in 70% ethanol (20 ml). Further adding the tuber extract dropwise at constant stirring, thus turning the mixture turbid. These culture bottles were then subjected to autoclaving at 121°C for 20 min at 15 lbs and then centrifuged. The obtained pellet of NPs was washed with water as well as ethanol thrice and then dried for 6 h.

Vinay et al. (2016) used bamboo culms for synthesizing silicon-based NPs. The inner portion of the stems of bamboo was used, which were chopped and pyrolyzed at 1250°C in the presence of argon gas at a high temperature for the bamboo to undergo self-thermochemical decomposition. This process was carried out for the removal of some of the organic compounds as well as water from the pieces of bamboo, which resulted in the charcoal bio template formation. For keeping the thermal stress at a minimum rate, the temperature was increased at 5°C/min. Then, the charcoal bio template was cooled to 200°C, naturally and further was removed post shutting off the inert atmosphere produced by argon.

Deshmukh et al. (2019) conducted the synthesis of boron-based NPs, that is, hexagonal boron nitride nanosheets (or h-BNNs). It involved a plant extract considering different plants, out of which the roots of *Panax ginseng*, leaves of *Morus nigra,* and stems of *Hovenia dulcis* were able to be used in the controlled experiment. This plant extract was dispersed in distilled water and further filtered, after which 10 mg h-boron nitride was added to it, ultimately sealing it. The material was then sonicated for a day at 40 kHz and 30°C, after which the thick nanosheets were settled. For separation of these h-BNNs, the supernatant underwent centrifugation at 15,000 rpm for an hour. The settled h- BNNs were dried and used for characterization. Thus, it was observed that the surfactant effect of phytocompounds was responsible for keeping the nanosheets dispersed. It was also noted that a high yield of h-BNN (around ∼23%) was obtained by this method of plant-mediated exfoliation.

Medina et al. (2019) prepared a stock of sodium tellurite in deionized water for the synthesis of tellurium based nanoparticles (TeNPs). Plant extract was made from fresh fruits particularly lime, orange, and lemon, which were squeezed, and the liquid was collected. At various metallic salt concentrations (like 25, 50, 75, and 100 × 10^−3^ mol/L) were added to a fixed volume of citric extract in a vial and were named depending on the extract used like orange-mediated TeNPs, lemon-mediated TeNPs and lime-mediated TeNPs. After getting mixed, a microwave-assisted reaction was carried out following which vials were placed inside the microwave and a single cycle of heating at 750 W was conducted for 10 s, observed *via* a cool-down of the reaction until room temperature.

## Applications

Use of metalloid nanoparticles has a wide range of applications such as delivery of potential drugs (Wang et al., 2007; Moghaddam et al., 2015), antibacterial applications (Fayaz et al., 2010), analysis of DNA, medical imaging (Amarnath et al., 2011), biosensors (Zheng et al., 2010) as well as tissue engineering (Kingsley et al., 2013).

### Antibacterial

Studies have proved the antibacterial features of silicon nanoparticles on both gram bacteria (-positive as well as -negative) (Smirnov et al., 2018). Previously, it has been shown (Ivanova et al., 2013; Hartley et al., 2015; Linklater et al., 2017) how the bactericidal effects of silicon-derived nanostructures were responsible for the mechanical damage of membranes of bacteria. Apart from this, the chemical impacts of indefinite silicon-derived NPs linked with the oxidative ramifications of silicon nanoparticles generating singlet oxygen as well as reactive oxygen species were considered (Kudryashov et al., 2018). Mechanism usually involved in antibacterial activity is photodynamic inactivation *via* O2, that is produced on the silicon-nanoparticle’s exterior during their production in water (Rioux et al., 2009) as well as alcohol (Xiao et al., 2011). This results in significant damage of biological objects, further leading to subsequent DNA damage. Additionally, Lin et al. (2012) examined that various tellurium-based nanomaterials like nanowires, nano pencils, nanorices, and nanocubes showed higher antibacterial activity against *E. coli* in comparison with silver nanoparticles. It was also proved that the antibacterial effects of nanocubes as well as nanorices are almost similar and their antibacterial effects are comparatively higher than nano pencils as well as nanowires. Besides this, Zare et al. (2012) illustrated the bactericidal activity of tellurium derived nanoparticles against various clinical segregates like *S. typhi, S. aureus*, *K. pneumoniae* and *P. aeruginosa* with minimum bactericidal concentration, ranging between 125 and 500 mg/L.

### Anticancer

The role of boron derived nanoparticles in cancer are highly varied. Arbab et al. (2006) examined their function in early diagnosis of breast cancer for detection of smaller breast cancers prior to their detection *via* traditional mammography. As studied by Kan-Dapaah et al. (2015), boron neutron capture therapy (BNCT) as well as photodynamic therapy are used in the case of adenocarcinoma of human lung’s A549 cells, resulting in conjugation along with a closo-dodecaborate, further leading to photoinduction of cell death. Apart from this, Sau and Rogach (2010) showed how BNCT has a vital function to play in human hepatoma, leading to dendritic glyco-borane which is responsible for around ten-fold improvement in finishing off the HepG2 cells of hepatoma. In addition to this, silicon-derived nanoparticles have major roles to play in cancer photothermotherapy in the case of pancreatic malignant tumor cells, as examined by Patra et al. (2015)- resulting in a high cytotoxic effect due to dimethyl sulfoxide porous silicon colloid combined with a near infrared (NIR) laser. In breast cancer cells, because of the covalent attachment of porphyrin to nontoxic porous silicon nanoparticles to the surface of breast cancer cells, it results in a photodynamic effect as a part of photodynamic therapy (Sperling and Parak, 2010). On the other hand, arsenic-derived nanoparticles contain various therapeutic applications with respect to cancer. Katz and Willne (2004) illustrated the potential therapeutic efficacy in human pancreatic cancer cells (PANC-1) as a result of their combination with anti-CD44v6 single chain antibody thus reducing the growth of tumor as well as enhanced apoptosis. Jain (2007) conducted a study on mouse melanoma tumors for studying photothermal cancer therapy, concluding that cadmium-tellurium nanoparticles have a great potential in cancer treatment. Targeted imaging as well as selective therapy for gastric cancer was studied by Jain (2008) who highlighted the role of high-performance HER2-RQDs nanoprobes *in-situ* gastric cancer. Baptista et al. (2011) signified the application in detection of micrometastases in early-stage breast cancer. Jianrong et al. (2004) illustrated the role of electrochemical DNA biosensor to detect chronic myelogenous leukemia (CML) with the use of nanostructured composites of cadmium-based telluride quantum dots, further distinguishing control samples of CML as positive and negative. Another study by Sekhon (2013) on the detection of prostate protein antigen (PSA) in prostate cancer was carried out, summarizing the use of cadmium-tellurium based nanotubes as an efficient alternative for clinical screening of cancer biomarkers like PSA. Loyd et al. (2005) proved how cadmium-tellurium derived quantum dots were a good fluorescence probe which were used to detect breast cancer cells and expression of HER2. Soflaei et al. (2012) analyzed the *in vitro* diagnosis of colon cancer which involved alteration of folic acid leading to gelatine plated cadmium-tellurium quantum dots to be a promising alternative for *in vitro* diagnosis of cancer. Treatment of melanoma cells with cadmium-tellurium quantum dots leads to the increase of stem-like cell subpopulations, thus proving an efficient therapeutic for cancer stem cells in case of melanoma (Cunha et al., 2009). These nanoparticles based on cadmium tellurium also have a role in photodynamic therapy for the treatment of HeLa contaminant KB cells with an enhanced photo cytotoxicity response (Xu et al., 2006). Applications of cadmium-tellurium derived nanocrystals involve hybrid materials with increased photodynamic properties for HepG2 and HeLa cancerous cells by improving the cell kill efficiency (Byrappa et al., 2008).

### Drug delivery

Out of the different types of metalloid NPs, specifically mesoporous silica-based NPs (MSNs) have been observed as a suitable candidate for drug delivery associated with targeting tumour cells because of their various benefits including good biocompatibility (Vallet-Regi et al., 2001). Basic silica NPs comprise unmasked negatively charged silanol groups at a pH of 7.4, and as a result associate firmly with erythrocytes, resulting in hemolysis (SlowingWu et al., 2009). Hemolytic activity of these NPs leads to a decrease of biocompatibility and affects their application with respect to passive tumour tissue targeting. Even though the primary reason for hemolysis is yet not completely understood, it has been verified that this activity can be inhibited by the incorporation of organic constituents in association with the silica framework, thus indicating that organosilica NPs sum up a probable drug delivery application (Urata et al., 2011; Chen et al., 2013; Teng et al., 2014).

Besides this, NPs stacked with meglumine antimoniate or nano antimony were used for the treatment of *Leishmania amazonensis* or *Leishmania infantum*. The outcomes of the study carried out by Sousa-Batista et al. (2019) showed the effective use of these NPs for improving the potency of meglumine antimoniate in visceral leishmaniasis treatment, manifesting their potential as a promising therapeutic strategy. Besides, this system has also a greater stability as compared to large liposomes. These nanoparticles were composed by double-emulsion solvent evaporation method as well as displaying a size ranging between 150 and 200 nm. BALB/c mice were contaminated with Leishmania and thus were used for accessing the above-mentioned biodistribution of nano antimony as well as meglumine antimoniate labelled nanoparticles.

### Antifungal

Growth of *A. solani* was significantly reduced with the help of MSNs (mesoporous silica-based nanoparticles) along with metalaxyl at different concentrations in comparison with the control one. The highest inhibitory percentage for the growth of *A. solani* was attained at around a concentration of 400 mg/L (Derbalah et al., 2018). The rate of this growth inhibition correlates with the concentration of MSNs. Both physical as well as structural properties of MSN like inflated surface area, distinctive structure, cylindrical shape, as well as consistent size of pores result in its high fungicidal efficacy against *A. solani*. Its accumulation in the membrane might also induce cell lysis (Gill et al., 2005) *via* the prevention of trans-membrane energy cycle, create insoluble compounds in fungus’s membrane which disrupts the electron transport chain, cause oxidation of cell membrane because of MSN’s positive charge as well as cell membrane’s negative charge which generates the electromagnetic attraction resulting in immediate cell death.

In addition to this, antifungal activities of biogenic tellurium-based nanoparticles were noted against *Candida albicans* (ATCC14053) *via* a sequential liquid dilution method, where the minimal inhibitory concentration was 100 μg/ml while the minimal fungicidal concentration was 2000 μg/ml (Zare et al., 2014). For investigating the effects of these nanoparticles along with squalene monooxygenase, the substrate amount was measured in both treated as well as nontreated fungal cells *via* reverse phase high performance liquid chromatography (R-HPLC). The effect of biogenic nanoparticles was also evaluated on the expression of squalene monooxygenase gene with the help of real-time polymerase chain reaction. They even inhibit the development of *C. albicans via* interaction with membrane sterols.

### Other applications

Boron based NPs (h-BNNs) have the potential to remove both anionic as well as cationic dyes as antioxidant agents *via* measurement of the radical scavenging activity and improvement of the mechanical properties of castor oil-based polyurethane composites. It was also noted that these boron-based nanosheets were an outstanding adsorbent in the case of environmental aspects (Lei et al., 2013; Lei et al., 2015). Low concentrations of silicon NPs have proven to improve the rate of seed germination in tomatoes (Siddiqui and Al-Whaibi, 2014). Suriyaprabha et al. (2012) showed that silicon NPs have also increased the rate of germination in seeds of maize by providing better uptake of nutrients and maintaining the pH as well as conductivity of the growth medium. Silicon based NPs on the seedlings of *Larix olgensis* showed that nanoparticles played a role in the improvement of seedling growth as well as quality, height, and diameter of root collar, lateral root number and length of seedlings as well as accelerated chlorophyll synthesis process. They increased the rate of seed germination as well as stimulated the antioxidant effect in case of salinity stress in squash (Siddiqui et al., 2014). The exogenous application of silica-based NPs showed an increase in seed germination of soybean seeds *via* enhancing nitrate reductase concentration as well as the ability of seeds for absorbing and utilizing water and nutrients (Zheng et al., 2005).

Savvas and Ntatsi (2015) showed how silica nanoparticles mitigated abiotic stress in vascular plants with silica deposition inside their tissues. Pretreating the seeds of *Calendula officinalis* L. with a concentration of 200 mg/L of these NPs led to the maximum increase of quercetin content in plants, which were grown under 50% field capacity (Rahimi et al., 2020). Treating the plant *Tanacetum parthenium* L. with around 25 × 10^–3^ mol/L, these NPs enhanced the *T. parthenium* germacrene A synthase, costunolide synthase, and *T. parthenium* (E)‐β‐caryophyllene synthase gene expression, which play a role in biosynthesis pathways of secondary metabolites, ‐caryophyllene as well as parthenolides, at a period of 6–24 h, along with having a similar impact on gene expressions exhibiting salinity stress (Khajavi et al., 2019). Besides, this, treatment of *Mentha piperita* L. with 50–100 mg/L silicon NPs increased the diameter as well as diameter of peltate glandular trichomes, chlorophyll contents, average photosynthetic rate, total phenolic and essential oil contents, and secondary metabolite content at 150 days post plantation (Ahmad et al., 2020). *Dracocephalum kotschyi* treated with 100 mg/L silica NPs post 2 days exposure time displayed an enhancement in biomass in comparison to the pronounced increase in pal and ras gene expression, along with an excellent rise in various secondary metabolites (Norouzi et al., 2019). Kalteh et al. (2014) stated that these NPs efficiently decreased the adverse impact of saline stresses on dry leaf fresh weight as well as chlorophyll content in *Ocimum basilicum* as well as enhanced proline levels, suggesting the induction of tolerance in basil plants.

## Conclusion

Bio-based techniques for the synthesis of metalloid NPs are still in their developmental phase and require stability, aggregation, and control of crystal growth, along with proper shape and size. Large-scale synthesis using bacteria, fungi, and plants seems interesting since it does not need any toxic as well as expensive chemical materials in order to produce stable NPs with well-defined sizes and shape. Further research is needed for the evaluation of their pharmaceutical potential. In future, it is expected that biogenic metalloid NPs might come out as an effective nanomedicine, individually, or in combination with FDA-approved drugs. Their applications as anticancer drugs in diagnostic oncology and cancer treatment have shown excellent results, due to their differentiation capabilities between normal and tumour cells. On the other hand, antibacterial and fungicidal activities also play vital roles in various important biological pathways in prokaryotes as well as eukaryotes. Targeted drug delivery has the potential of reducing side effects which may harm normal organs as well as tissues by facilitating the accumulation of drug at the tumour site. In future various other bacterial, fungal and plants species can be utilized for the synthesis of different types of metalloid nanoparticles. They can be utilized due to their multifunctional nanocarriers for various targeting strategies involving passive and active targeting as well as magnetic field directed targeting. It is anticipated that such nanoparticle systems might improve the potential of advanced diagnostics as well as treatment strategies in coming future which leads to the improvement of both the duration as well as the quality of a patient’s life.
